# Being a Parent Together: Parental Role Salience Promotes an Interdependent Self-Construal

**DOI:** 10.3389/fpsyg.2018.01462

**Published:** 2018-08-15

**Authors:** Yuanyuan Jamie Li, Han Gong

**Affiliations:** ^1^Department of Marketing & E-business, School of Business, Nanjing University, Nanjing, China; ^2^Department of Marketing, College of Business, Shanghai University of Finance and Economics, Shanghai, China

**Keywords:** parenthood, role priming, self-construal, interdependence, independence

## Abstract

Self-construal has been shown to be exert consequential influences on thinking and doing. Although how people construe themselves is often deemed as a chronic and stable individual difference, relatively little is known about the factors that could potentially shape the extent to which individuals form an independent-self or an interdependent-self. In the current work, we try to explore whether and how the salience of parental roles would affect self-construal. Given that an interdependent self-construal helps individuals maintain connectedness and harmony with others in a group, which is adaptive for being a parent, we propose that parental roles tend to increase the perceived connection with others, thus leading to an interdependent self-construal. Findings from three studies consistently show that a salient parental role promotes an interdependent self-construal. Moreover, we observe that parents’ role salience only prompts an interdependent self-construal in relation to other people without increasing the connection with one’s future self. Theoretical and practical implications of our findings are discussed.

## Introduction

Unlike most other species, human offspring is born small and vulnerable, and needs long time to mature (Preston, 2013). Cárdenas et al. (2013) have proposed that human infants and children are the most costly to rear among primate infants. For example, the average cost for parents to raise up one single child in United States is between $205,960 and $475,680, without considering college tuition (Lino, 2010). Besides financial resources, people also have to spend tremendous amount of time and effort to take care of offsprings (Griskevicius and Kenrick, 2013). Indeed, children without parents are less likely to survive and have substantially lower reproductive fitness (Hill and Hurtado, 1997; Geary, 2000; Taylor et al., 2000).

To ensure the survival of offspring, caregiving behaviors have evolved. Caregiving is to provide support and protection to those who are chronically dependent or temporally in need (Bowlby, 1988; Solomon and George, 1996). The caregiving motive guarantees the survival and development of individuals in need by facilitating nurturing behaviors (Glocker et al., 2009; Sherman et al., 2013), which are manifested by carefulness (Sherman et al., 2009), protection (Daly and Wilson, 1988), and providing support to others (Smith et al., 1987). In general, parents are major caregivers for dependent human infants.

Given that the priority of parental role is to ensure the adaptation of offspring (Griskevicius and Kenrick, 2013), in light of a salient parental role, behavioral changes to provide offspring protection, such as more trusting of others, increased nurturance and giving without expectations of reciprocation, are therefore expected. Indeed, previous literature has found both correlational and causal relationships between parenthood and risk-vigilant perceptions (Solomon and George, 1996; Wang et al., 2009; Cameron et al., 2010). Specifically, the salience of parental role increases risk perception and risk averse choices among parents (Eibach and Mock, 2011). Besides risk-vigilant attitudes, the salience of parental role has been found to expand the domain of moral judgments (Eibach et al., 2009). However, it is still an open question whether and how parental role salience influences individuals’ perception in relation with others.

Malleable self theory proposes that a subset of working self-concepts may be activated, and we may define ourselves accordingly (Markus and Kunda, 1986). Individuals may construe themselves differently in relation to the collective — interdependent vs. independent self-construals (Markus and Kitayama, 1991). As Singelis (1994) posits, these two images of self either emphasize the connectedness and interpersonal relations (i.e., interdependent self-construal) or separateness and uniqueness of individuals (i.e., independent self-construal). An interdependent self-construal concerns individuals’ belief about their connectedness with others, highlighting external features, belonging, “reading others’ minds,” and commitment to duties and obligations; while an independent self-construal emphasizes internal ability, unique self, direct communication and a focus on self-interest (Markus and Kitayama, 1991; Singelis, 1994; Kitayama et al., 2017).

Most previous research has focused on the consequences of self-construal on human behaviors. For example, priming an interdependent self-construal (vs. independent self-construal) has been shown to increase the attention directed toward preventing losses (Aaker and Lee, 2001) and social norms (Ybarra and Trafimow, 1998). An interdependent self-construal (vs. independent self-construal) has been demonstrated to be an effective way to promote cooperation in social dilemma (Utz, 2004). Mandel (2003) also shows that activating an interdependent self-construal (vs. independent self-construal) increases consumers’ financial risk-taking, and that the size of social network is one of the mediators. These findings are consistent with the original definition of self-construal that an interdependent self-construal emphasizes on connectedness with others, and relying on others in times of need.

However, relatively little research has explored the antecedents of interdependent or independent self-construals. According to prior literature, the two self-construals originate from two different cultures: the interdependent self-construal is nurtured in Eastern cultures whereas the independent self-construal is more rooted in Western cultures (Markus and Kitayama, 1991). Although interdependent and independent self-construals are chronically fostered by cultures, they can also be experimentally manipulated to be temporarily more salient (Aaker and Lee, 2001; Mandel, 2003; Hong and Chang, 2015). For example, by directing people’s attention to either singular (e.g., I, my, me) or plural (e.g., we, our, us) pronouns, an independent self-construal or an interdependent self-construal can be made more accessible accordingly (Hong and Chang, 2015). Yet, despite the importance of self-construal, not enough work has been conducted to better understand the antecedents of interdependent or independent self-construals. Our project aims to shed some more light by showing that other factors may temporarily activate an interdependent self-construal, such as the salience of parenting role.

As social animals, human beings obtain what they need from the society (Zhou et al., 2009). Since it is extremely costly to raise children, behaviors that help caregivers/parents obtain resources from the social system are evolutionarily adaptive. When parental role becomes salient, it is beneficial to get connected with others and focus on interpersonal relations (Markus and Kitayama, 1991; Singelis, 1994). Therefore, from an evolutionary perspective, staying together and taking care of offspring in a social environment gives parents a better chance of successfully raising their children (Preston, 2013). Moreover, the salience parental role increases caregiving-related responsibility (Eibach et al., 2009). Caregiving-related responsibility directs attentions to others’ welfare and distress, thus other-focused and connected (Mikulincer et al., 2005). Given that individuals high on interdependent self-construal are more likely to commit to duties and obligations (Kitayama et al., 2017), it seems plausible to hypothesize that parental role salience may boost an interdependent self-construal. Distinct from prior work examining the downstream consequences of self-construals, the current paper contributes to the literature on self-construal by investigating a potential antecedent – the salience of parental role. Specifically, we aim to explore whether and how parental role salience would influence the way individuals construe themselves.

To demonstrate our proposed hypothesis that parental role salience increases an interdependent (vs. independent) self-construal, three empirical studies were conducted. In Study 1, a correlational study was conducted to explore the relationship between parental role salience and an interdependent self-construal. Furthermore, we established the causal relationship between parental role salience and an interdependent self-construal by manipulating the salience of parental role in Study 2A and Study 2B. In Study 2A, participants indicated their interdependent self-construal after viewing either parental care products or office products. Study 2B theoretically replicated the findings in Study 2A with another parental role salience manipulation. Moreover, to exclude the possibility that parental role salience increases connectedness in general, the impact of parental role salience on connectedness with both the others and future self were explored.

## Study 1

To test the hypothesis that parental role salience promotes an interdependence self-construal, we first conducted a correlational study, where parental care motivation and self-construal were both measured (Singelis, 1994). We expected that individuals with higher parental care motivation would be more likely to have an interdependent construal of self.

### Method

#### Participants

Written informed consent was obtained from the participants across all three studies. All studies were carried out in accordance with the recommendations of APA’s ethical guidelines, Research Ethical Committee of Business School, Nanjing University. The protocol was approved by the Research Ethical Committee of Business School, Nanjing University. Two hundred one participants (*M*_age_ = 34.28, *SD*_age_ = 10.72; 118 men; 83 nonparents) located in the United States were recruited from Amazon’s Mechanical Turk (MTurk) for monetary compensation.

#### Materials and Procedure

##### Parental Care and Tenderness questionnaire (PCAT)

The PCAT questionnaire (Buckels et al., 2015) was used to measure individuals’ activation of the parental care motivational system. The 25-item scale contains five dimensions — liking of children (five items; e.g., “I think that kids are annoying (reverse-coded),” anchored by 1 = “strong disagree” and 5 = “strongly agree”), protective impulses for children (five items; e.g., “I would use any means necessary to protect a child, even if I had to hurt others,” anchored by 1 = “strong disagree” and 5 = “strongly agree”), caring for children (five items; e.g., “Babies melt my heart,” anchored by 1 = “strong disagree” and 5 = “strongly agree”), tenderness aroused by generally positive parenting-related stimuli (five items; e.g. “You see a father tossing his giggling baby up into the air as a game,” anchored by 1 = “no tenderness at all” and 5 = “a lot of tenderness”) and tenderness aroused by negative parenting-related stimuli (five items; e.g., “You see that a baby is sick,” anchored by 1 = “no tenderness at all” and 5 = “a lot of tenderness”). The higher of the score in these five dimensions indicates a stronger motivation to parental care, and a more salience of parental role.

##### Independent and interdependent self-construal measure

We used the scale developed by Singelis (1994), which consists of 24 items, half measuring an independent self-construal (e.g., “I enjoy being unique and different from others in many respects”) and the other half an interdependent self-construal (e.g., “It is important for me to maintain harmony within my group”). Participants were asked to indicate the extent to which they agreed with each statement (1 = “strongly disagree,” 7 = “strongly agree”). The higher of the score on each dimension, the more interdepend and independent they are, respectively.

All participants first completed the PCAT questionnaire and then proceeded to the Independent and Interdependent Self-Construal measure. Demographic information was collected at the end of the study.

#### Results and Discussion

SPSS was applied to analyze the data across all three studies. All scales yielded high reliability (all Cronbach’s alphas > 0.80). Correlational analyses between PCAT and Self-Construal measure revealed that individuals with a higher activation of parental care motivation (*M* = 3.77, *SD* = 0.64) had a significantly higher interdependent self-construal (*M* = 5.35, *SD* = 0.88; *r* = 0.49, *p* < 0.001), as well as an independent self-construal (*M* = 5.03, *SD* = 0.94; *r* = 0.42, *p* < 0.001, see **Table 1** for details). Moreover, one-way ANOVA was used to check whether parental status had any influences on interdependent self-construal. The result showed that participants who are parents tended to have a significantly higher activation of the parental care motivational system, as compared with those who are not parents, *F*(1,199) = 17.45, *p* < 0.001, η^2^ = 0.08. More importantly, being a parent also lead to a higher interdependent self-construal, *F*(1,199) = 13.24, *p* < 0.001, η^2^ = 0.06, but not a higher independent self-construal, *F*(1,199) = 2.59, *p* > 0.1. These correlational findings are consistent with our hypothesis that there is a positive relationship between parental role salience and an interdependent (vs. independent) self-construal.

**Table 1:** Correlations between PCAT, interdependent self-construal, and independent self-construal.

	Parents	Like	Protect	Care	Tender_positive	Tender_negative	Interdependence	Independence
PCAT	0.28^∗∗^	0.56^∗∗^	0.71^∗∗^	0.82^∗∗^	0.78^∗∗^	0.69^∗∗^	0.49^∗∗^	0.42^∗∗^
Like	0.13	1.00	0.17^∗^	0.27^∗∗^	0.32^∗∗^	0.14	−0.05	−0.08
Protect	0.17^∗^	0.17^∗^	1.00	0.49^∗∗^	0.59^∗∗^	0.35^∗∗^	0.42^∗^	0.48^∗∗^
Care	0.31^∗∗^	0.27^∗∗^	0.49^∗∗^	1.00	0.58^∗∗^	0.59^∗∗^	0.56^∗∗^	0.42^∗∗^
Tender_positive	0.09	0.32^∗∗^	0.59^∗∗^	0.58^∗∗^	1.00	0.35^∗∗^	0.35^∗∗^	0.37^∗∗^
Tender_negative	0.29^∗∗^	0.14	0.35^∗∗^	0.59^∗∗^	0.35^∗∗^	1.00	0.51^∗∗^	0.34^∗∗^
Independence	0.25^∗∗^	−0.05	0.42^∗∗^	0.56^∗∗^	0.35^∗∗^	0.51^∗∗^	1.00	0.57^∗∗^
Interdependence	0.11	−0.08	0.48^∗∗^	0.42^∗∗^	0.37^∗∗^	0.34^∗∗^	0.51^∗∗^	1.00

*^∗^Correlation is significant at the 0.05 level (2-tailed). ^∗∗^ Correlation is significant at the 0.01 level (2-tailed).*

If parental role salience leads to a higher interdependent self-construal, parental status should moderate the relationship between parental care motivation and the level of interdependent self-construal. Specifically, parents with stronger parental care motivation should have the highest level of interdependent self-construal. To explore the moderation effect of parental role, Model 1 of process analysis (Hayes, 2013) was applied. As recommended by Hayes (2013), we set the moderation model using 5000 bootstrap iterations and 95% confidence intervals, with PCAT (centered) as independent variable, parental status as moderator (parent = 1; nonparent = 0), and interdependent self-construal as dependent variable. The model was significant, *R*^2^ = 0.28, *F*(3,197) = 25.77, *p* < 0.001. Both PCAT (*b* = 0.86, *t* = 7.33, *p* < 0.001) and parental status (*b* = 0.23, *t* = 1.90, *p* = 0.056) were significant predictors for the level of interdependent self-construal. Both the status of being a parent and a strong parental care motivation were positively related to a higher level of interdependent self-construal. Importantly, the interaction between PCAT and parental status was significant as well, *b* = −0.49, *t* = −2.62, *p* < 0.01. Among nonparents, higher PCAT individuals also had a significant higher interdependent self-construal, effect = 0.86, *t* = 7.33, *p* < 0.001 (0.4054, 0.7036). Moreover, among parents, higher PCAT individuals had a significant higher interdependent self-construal, effect = 0.37, *t* = 2.53, *p* < 0.05 (0.0525, 0.4235).

Similar to interdependent self-construal, the same moderation analysis was applied to independent self-construal. PCAT was a significant predictor for an independent self-construal as well, *b* = 0.62, *t* = 5.28, *p* < 0.001, suggesting that individuals with a stronger parental care motivation had a higher level of independent self-construal as well. On the contrary, being a parent did not have a higher independent self-construal, *t* < 1. Importantly, the interaction between PCAT and parental role was far from significant, *t* < 1.

Study 1 provided the preliminary evidence that the salience of parental role, as manifested by the status of being a parental and the activation of parental care motivation, was significantly correlated with an interdependent self-construal. The interaction between parental status and PCAT suggested that the parental care motivation is associated with a higher level of interdependent self-construal, especially among nonparents. Although parental care motivation was positively correlated with both chronically interdependent and independent self-construals, and the two construals of self may coexist within individuals (Mandel, 2003), parents only had significantly higher level of interdependent self-construal, not independent self-construal. Our correlational findings indicate that parenting, illustrated by both parental role status and parental care motivation, intertwined with interdependent self-construal. Therefore, in Studies 2A and 2B, we plan to examine the causal relationship between parental role salience and an interdependent self-construal.

## Study 2A

Study 1 demonstrated a significant positive correlation between parental role, the activation of parental care motivational system and an interdependent self-construal. In Studies 2A and 2B, we experimentally manipulated the salience of parental role, and expected to show that parental role salience would enhance the connectedness between self and others (i.e., an interdependent self-construal).

### Method

#### Participants

One hundred participants (*M*_age_ = 36.03, *SD*_age_ = 14.63; 45 men; 41 nonparents) located in the United States were recruited from MTurk for monetary compensation.

#### Materials and Procedure

We used a 2 (parental role manipulation: salience vs. control) × 2 (parental status measure: Yes or No) between-subjects design. First, all participants were invited to use three pictures to compose a story. Adapted from Griskevicius and Kenrick (2013), half of participants were provided with pictures involving parental care products (i.e., stroller, milk bottle, and shower bathtub) and the other half with pictures of office products (i.e., pen, eraser, and sticker). After the parental role manipulation, all participants were instructed to indicate their perceived connectedness with other people in general on a 100-point scale (Bartels and Urminsky, 2011), where 0 means “completely disconnected” and 100 means “completely connected.” At the end of the study, all participants reported their age, gender, and parental status.

#### Results and Discussion

A 2 × 2 ANCOVA was conducted, with parental role salience and parental status as two between-subject factors and gender as a covariate^1^. Gender was not a significant predictor for an interdependent self-construal, *F* < 1. The main effect of parental role salience was not significant as well, *F* < 1. Consistent with the findings of Study 1, the main effect of parental status was significant, *F*(1,95) = 4.57, *p* < 0.05, η^2^ = 0.046. Specifically, parents (*N* = 59; *M* = 60.85, *SD* = 29.52) had a significantly higher level of interdependent self-construal than nonparent (*N* = 41; *M* = 46.95, *SD* = 32.54). Importantly, this main effect of parental role was qualified by a marginally significant interaction with parental role salience manipulation, *F*(1,95) = 3.04, *p* = 0.08, η^2^ = 0.031. Specifically, reminding parents of their parental role (*M*_priming_parent_ = 66.71, *SD* = 29.13) significantly increased interdependent self-construals than parents in nonsalient parental role condition (*M*_control_parent_ = 52.29, *SD* = 28.55), *F*(1,95) = 3.23, *p* = 0.076, η^2^ = 0.033. However, the salience of parental role did not influence interdependent self-construals among nonparents (*F* < 1; *M*_priming_nonparent_ = 43.53, *SD* = 33.30 vs. *M*_control_nonparent_ = 49.38, *SD* = 32.48). Another perspective on the interaction between parental role salience and parental status suggests that parents have a higher level of interdependent self-construal only when their parental roles become salient, *F*(1,95) = 7.18, *p* < 0.01, η^2^ = 0.07. However, in the control condition parents and nonparents did not differ in terms of the interdependent self-construal level (*F* < 1). These results suggest that experimentally induced parental role salience may only lead to higher levels of interdependent self-construal among parents.

## Study 2B

In Study 2B, we used a different method to manipulate parental role salience, which serves as a conceptual replication of previous studies. Additionally, to explore whether parental role salience changes construal level in general, connection with both the others and future self were measured in Study 2B. If parental role salience changed interdependent self-construal by construe everything closer (Trope et al., 2007), we should observe a stronger perceived connection with future self as well. Otherwise, our findings are specific to connection with others.

### Method

#### Participants

One hundred ninety-six participants (*M*_age_ = 35.53, *SD*_age_ = 12.29; 88 men; 74 nonparents) from the United States were recruited from MTurk for monetary compensation.

#### Materials and Procedure

This is a 2 (parental role: salient vs. control) × 2 (parental status: parents vs. nonparents) × 2 (connection target: future self vs. other) mixed design, with parental role and parental status as between-subject factors, and connection target as a within-subject factor.

First, participants were randomly assigned to one of the two parental role manipulation conditions. The salience of parental role was manipulated by placing the dependent measures either after (parental role salient condition) or before (control condition) the parenthood-related questions: (1) whether they were parents, (2) how many children they had, and (3) how old their youngest child was. Participants who reported their parenthood-related questions at the beginning had a stronger parental role salience as compared with those who reported parenthood-related questions after dependent variables (Eibach et al., 2009; Eibach and Mock, 2011). Connections with others and future self were measured in the same way scale as in Study 2A (Bartels and Urminsky, 2011). At last, all participants reported their age and gender.

#### Results and Discussion

**FIGURE 1:**
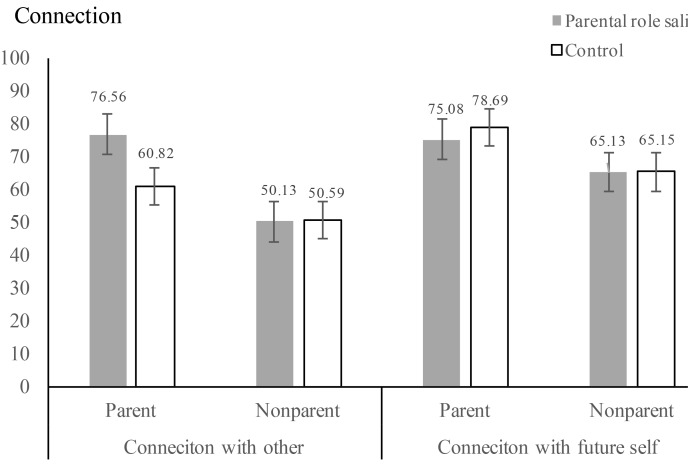
Average connection across all conditions.

We conducted a 2 (parental role) × 2 (parental status) × 2 (connection target) repeated-measures analysis, with parental role salience and parental status as between-subjects factors, and connection with others vs. future self as the within-subjects factor^2^. Consistent with previous findings, there was a significant main effect of parental status, *F*(1,192) = 29.37, *p* < 0.001, η^2^ = 0.13. In general, parents (*M* = 72.79, *SD* = 17.98) reported to have significantly higher connections with the others and with the future self as compared with nonparents (*M* = 57.74, *SD* = 20.22). This main effect was qualified by a significant three–way interaction, *F*(1,192) = 6.09, *p* = 0.014, η^2^ = 0.03. Specifically, for participants who are parents, parental role salient manipulation (vs. control condition) made participants to perceive a higher connection with the others [*F*(1,192) = 12.02, *p* < 0.01, η^2^ = 0.06; *M*_salience_ = 76.56, *SD* = 22.24 vs. *M*_control_ = 60.82, *SD* = 24.43], but not with the future self (*F* < 1; *M*_salience_ = 75.08, *SD*_salience_ = 20.69 vs. *M*_control_ = 78.69, *SD*_control_ = 19.69). However, among nonparents, neither did parental role salience manipulation influence the connections with the others (*F* < 1; *M*_salience_ = 50.13, *SD*_salience_ = 28.83 vs. *M*_control_ = 50.59, *SD*_control_ = 26.28) nor the future self (*F* < 1; *M*_salience_ = 65.13, *SD*_salience_ = 23.71 vs. *M*_control_ = 65.15, *SD*_control_ = 21.23; see **Figure 1** for details). Study 2B replicated the findings of Study 2A with another parental role manipulation. Again, we found that parental role salience increased perceived connection with others, and a higher level of interdependent self-construal. Our findings about felt connection with future excluded the possibility that parental role salience increased construal level and decreased distance with everything (Trope et al., 2007).

## General Discussion

Across three empirical studies, we showed that the salience of parental role increased the perceived connection with the others, namely a higher level of interdependent self-construal. In Study 1, correlational findings suggest that participants who are parents or high in parental care motivation have a higher level of interdependent self-construal than those who are nonparents or low in parental care motivation. Study 2A illustrated that temporarily highlighting the salience of parental role among parents increased their perceived connectedness with others. Study 2B replicated these findings with a different manipulation of parental role salience and also ruled out a possible explanation that parental role salience increases a general connection with other beings including one’s future self.

Cooperative breeding system is a breeding system in which all group members of a given society help genetic parents to rear their offspring (Hrdy, 2007). Such a system has been argued to be very adaptive for human beings, as it increases the survival rate of offspring. This is consistent with what we found that parental role salience increased interdependent self-construal. High level of interdependent self-construal is adaptive for parenting. First, parenting is a process to provide care to dependent others, which implies a shift of focus from the self to others. Individuals with a high level of interdependent self-construal pay more attention onto others, which is consistent with parenting-related goals (Hong and Chang, 2015). Second, as social animals, staying together with ingroups enables individuals to receive more resources from the society, like safety and social support (Baumeister and Leary, 1995; Preston, 2013). This is of great importance for individuals’ survival and parenting goal. Third, an interdependent self-construal emphasizes personal relationship and commitment to duties and obligations (Markus and Kitayama, 1991; Kitayama et al., 2017). Taking care of offspring is extremely resource consuming and full of duties and obligations (Lino, 2010). Therefore, salient interdependent self-construal helps parents better take care of offsprings when their parenting role become salient.

The findings of our paper also have important theoretical contributions. As reviewed in the introduction, previous literature only focuses on the consequences of interdependent/independent self-construals, while viewing self-construals as a chronic cultural underpinning. Although interdependent or independent self-construals could become temporarily more accessible through priming, it is still a missing point what factors could serve as the antecedents of self-construals in addition to cultural factors. This paper fills this gap by showing that parental role salience is one of the factors that lead to a heightened level of interdependent self-construals. Future work may explore other potential antecedents of one’s self-construal.

There are several limitations for this paper. First, all participants in the paper were recruited online from United States population. It is still an open question whether the same effect could be replicated among other population in different cultures. Second, we only used a survey and two experiments to illustrate the relationship between parental role salience and interdependent self-construal in this paper. Findings from other methodologies, such as secondary data, field experiments could increase the generalizability of our findings. Third, future work could explore and show the exact mechanisms underlying our findings. In sum, the generalizability of our findings could be investigated through multi-method among diverse population in the future.

## Author Contributions

YL contributed to the conception of the work, data acquisition, data analysis, and data interpretation, as well as drafting and revising the manuscript. HG contributed to the conception of the work, data analysis, data interpretation, as well as revising the manuscript. All authors approved the final version.

## Conflict of Interest Statement

The authors declare that the research was conducted in the absence of any commercial or financial relationships that could be construed as a potential conflict of interest.

## Footnotes

1 Participants in the parental role salient condition did not differ from those in the control condition on demographics: *F*(1,98)_age_ = 1.10, *p* = 0.30, *F*(1,98)_gender_ = 2.04, *p* = 0.16, *F*(1,98)_income_ = 0.02, *p* = 0.89, *F*(1,98)_employment_ = 0.08, *p* = 0.78. Additionally, parents did not differ from nonparents: *F*(1,98)_age_ = 0.77, *p* = 0.38, *F*(1,98)_gender_ = 0.49, *p* = 0.49, *F*(1,98)_income_ = 0.44, *p* = 0.51, *F*(1,98)_employment_ = 2.34, *p* = 0.13.

2 Participants in the parental role salient condition did not differ from those in the control condition on demographics as well: *F*(1,193)_age_ = 0.00, *p* = 0.98, *F*(1,194)_gender_ = 1.10, *p* = 0.30, *F*(1,194)_income_ = 0.09, *p* = 0.77, *F*(1,194)_employment_ = 1.95, *p* = 0.16. Additionally, parents did not differ from nonparents, except for age: *F*(1,193)_age_ = 29.36, *p <* 0.001, *F*(1,194)_gender_ = 0.67, *p* = 0.41, *F*(1,194)_income_ = 0.00, *p* = 0.97, *F*(1,194)_employment_ = 0.50, *p* = 0.48. It is reasonable that parents are older than nonparents, which did not interfere with our findings, as the interaction between parental role salient manipulation and parental status did not interact significantly, *F*(1,191) = 0.78, *p* = 0.38.
